# Identification of key pathways and genes that regulate cashmere development in cashmere goats mediated by exogenous melatonin

**DOI:** 10.3389/fvets.2022.993773

**Published:** 2022-09-29

**Authors:** Zhihong Liu, Zhichen Liu, Qing Mu, Meng Zhao, Ting Cai, Yuchun Xie, Cun Zhao, Qing Qin, Chongyan Zhang, Xiaolong Xu, Mingxi Lan, Yanjun Zhang, Rui Su, Zhiying Wang, Ruijun Wang, Zhixin Wang, Jinquan Li, Yanhong Zhao

**Affiliations:** ^1^College of Animal Science, Inner Mongolia Agricultural University, Hohhot, China; ^2^Key Laboratory of Animal Genetics, Breeding and Reproduction, Inner Mongolia Agricultural University, Hohhot, China; ^3^Key Laboratory of Mutton Sheep Genetics and Breeding, Ministry of Agriculture, Hohhot, China; ^4^Goat Genetics and Breeding Engineering Technology Research Center, Inner Mongolia Agricultural University, Hohhot, China; ^5^Inner Mongolia Autonomous Region Agriculture and Animal Husbandry Technology Extension Center, Hohhot, China; ^6^Inner Mongolia Academy of Agricultural and Animal Husbandry Sciences, Hohhot, China; ^7^Hebei Normal University of Science and Technology, Qinhuangdao, China

**Keywords:** melatonin, WGCNA, hair follicle cycle, Inner Mongolia cashmere goats, PDGF

## Abstract

The growth of secondary hair follicles in cashmere goats follows a seasonal cycle. Melatonin can regulate the cycle of cashmere growth. In this study, melatonin was implanted into live cashmere goats. After skin samples were collected, transcriptome sequencing and histological section observation were performed, and weighted gene co-expression network analysis (WGCNA) was used to identify key genes and establish an interaction network. A total of 14 co-expression modules were defined by WGCNA, and combined with previous analysis results, it was found that the blue module was related to the cycle of cashmere growth after melatonin implantation. Kyoto Encyclopedia of Genes and Genomes (KEGG) pathway enrichment analysis showed that the first initiation of exogenous melatonin-mediated cashmere development was related mainly to the signaling pathway regulating stem cell pluripotency and to the Hippo, TGF-beta and MAPK signaling pathways. *Via* combined differential gene expression analyses, 6 hub genes were identified: *PDGFRA, WNT5A, PPP2R1A, BMPR2, BMPR1A*, and *SMAD1*. This study provides a foundation for further research on the mechanism by which melatonin regulates cashmere growth.

## Introduction

The Inner Mongolia cashmere goat is an economically important animal with diverse uses and is an important economic breeding stock in China. Cashmere is one of the finest and lightest fibers produced by animals; it is noted for its outstanding properties and rarity and is much more expensive than wool of the same fineness. It is used especially in the production of high-end textiles. Cashmere is produced by hair follicles, an accessory organ of the skin (1, 2). Cashmere goat hair can be divided into two types of fibers: unmedullated cashmere and well-medullated coarse hair. Hair follicles can also be divided into primary hair follicles and secondary hair follicles (3). In Inner Mongolia cashmere goats, cashmere growth exhibits a seasonal pattern and a periodic change influenced by the natural photoperiod (2). Each year, cashmere starts growing in late summer, stops growing in the middle of winter and falls off naturally in spring. These periodic changes occur through three phases of cellular activity: growth, regression, and repose (4). Secondary hair follicles grow from April to November, regress from December to January, and rest from February to March.

The growth cycle of cashmere is affected by many factors, such as sunshine duration, melatonin (MT; N-acetyl-5-methoxy-tryptamine), nutrition, genetics, endocrine status, etc. (5). MT is an endogenous hormone produced by the pineal gland. Its secretion has a distinct circadian rhythm, with inhibition during the day and active secretion at night. MT has long been recognized as an effective regulatory neuroendocrine substance associated with hair growth and the hair cycle, dependent on photoperiods, seasonal rhythms and environmental factors. Notably, MT performs unique biological functions in regulating hair growth in goats (6). Studies have shown that MT is a key mediator between the photoperiod and cashmere growth and that the level of circulating MT directly affects cashmere growth. Exogenous MT has been confirmed to have a positive effect on cashmere growth. In the non-growing period, the use of exogenous MT can stimulate the growth of cashmere but can also lead to early shedding of cashmere followed by another cashmere growth period (7). Considering the above effects of MT, we sought to determine whether the economic benefit of cashmere goats can be increased by artificially implanting MT to change the reception of light signals. Our previous study showed that continuous MT implantation promoted the entry of cashmere into hair follicles 2 months in advance of the seasonal cycle and promoted the development of secondary hair follicles. MT significantly affected the expression of *WNT10B*, β*-catenin* and other proteins in the skin tissue of Inner Mongolia cashmere goats. With the rapid development of high-throughput sequencing technology, through the analysis of differentially expressed genes (DEGs) and functional enrichment analysis, scientists have identified many regulatory factors and signaling pathways that may influence the hair follicle cycle: the WNT signaling pathway, fibroblast growth factor (FGF) family, bone morphogenetic protein (BMP) family, Sonic hedgehog (SHH) signaling pathway, transforming growth factor (TGF) family, Notch signaling pathway, etc. (8–10).

Bioinformatics methods are increasingly used for the exploration and analysis of target genes or proteins (11). Weighted gene co-expression network analysis (WGCNA) is a method used to study gene modules related to traits at the whole-transcriptome level. This mathematical method is used to transform expression data into scale-free distribution network information and then perform clustering on the target gene set and identify the gene modules of interest. In contrast to traditional gene clustering approaches, WGCNA clustering can reveal biological significance and has been widely used in gene mining, gene function prediction and other aspects. Regarding WGCNA in cashmere goats, a research group found through WGCNA that *WNT10A* is a key gene in the early stage of development and maturation of fetal skin hair follicles in Inner Mongolia cashmere goats (12). To date, WGCNA has been used in many studies on diseases, especially tumors (13–16), but the use of WGCNA to evaluate the effects of MT implantation in Inner Mongolia cashmere goats has not been reported. Therefore, to explore the genes and pathways through which MT promotes early growth of cashmere, we conducted this experiment.

## Materials and methods

### Sample collection

The research samples were collected from the Inner Mongolia White Cashmere Goat Breeding Farm in Etoke Banner, Ordos City, Inner Mongolia. 61-year-old ewes with similar body weights, with no relation to each other and in good growth condition were selected and divided into two groups. One group was implanted with MT, and the other group was used as a control. In the implanted group, MT was implanted subcutaneously behind the ears of the cashmere goats at a dose of 2 mg/kg BW every 2 months. Skin samples of 1 cm × 1 cm were collected from the scapula side of the ewes at the beginning of each month, and the skin samples were stored in an ultralow temperature refrigerator at −80°C for later use.

### Preparation of frozen sections and HE staining

Spare tissue samples were removed from −80°C, thawed in equilibrium at 4°C, placed in 4% paraformaldehyde solution and fixed at 4°C overnight. The next day, the fixed tissues were washed three times with PBS for 3 min each time. After the filter paper dried, the tissues were placed in 30% sucrose solution and dehydrated overnight at 4°C until the tissues sank to the bottom. The excess water was absorbed with filter paper, the appropriate angle was adjusted according to the sectioning direction, and the tissue was placed on the precooled section substrate; then, the tissues were embedded with OTC embedding agent before cryosectioning. The microtome was prechilled, and the tissues were sectioned after being completely frozen. The sections were stained using a hematoxylin eosin (HE) staining kit (G1120, Solarbio) according to the instructions.

### Total RNA extraction from skin

Total RNA was extracted from the skin of three cashmere goats using an RNAiso Plus Kit (TRIzol method). The purity and integrity of total RNA were measured using a sterile UV–VIS spectrophotometer and an Agilent 2,100 bioanalyzer, respectively, and the three RNA samples were then mixed. Total RNA was stored in a freezer at −80°C.

### Construction of the sequencing library

The cDNA library for transcriptome sequencing was constructed according to the operating instructions of an Illumina TruSeqTM RNA Sample Preparation Kit. Total RNA extracted from three cashmere goats per group in each month was mixed together in equal amounts. The mRNA was purified with oligo(dT) magnetic beads and sheared into 100–400 bp mRNA fragments. Double-stranded cDNA was synthesized using the mRNA fragments as templates, an exonuclease and a polymerase. To obtain the sequencing library, the double-stranded cDNA was phosphorylated, ligated to sequencing adapters and subjected to poly(A) tailing prior to PCR amplification.

### Transcriptome sequencing and splicing

The Illumina HiSeqTM 2000 sequencing platform was used for paired-end sequencing of cDNA. A 2 × 100 bp sequencing protocol was used for sequencing by Shanghai Meggie Biopharmaceutical Co., Ltd. After sequencing, referenced and unreferenced genomes were used to compare the reads, and assembly was conducted with the Trinity and Velvet methods. The filtered reads were aligned to the goat (*Capra hircus*) genome. The FPKM value of each gene was calculated to estimate the gene expression level.

### Construction of the gene co-expression network

Before WGCNA, the selected gene set was screened and filtered to remove low-quality genes or samples with unstable effects on the results to improve the accuracy of network construction. A weighted gene co-expression network was constructed using WGCNA (V. 147) package in R software (17). The Pearson linear correlation coefficient of each pair of genes in the gene set was calculated to construct the correlation coefficient matrix (*S*_*ij*_ = |*cor*(*X*_*i*_, *X*_*j*_)|). To remove the influence of low correlation coefficients, an appropriate threshold (β) was selected for exponential weighting of the correlation coefficient matrix and construction of the adjacency matrix. Then, the adjacency matrix was transformed into the topological overlap matrix ($TOM_{ij}=\frac{l_{ij}+a_{ij}}{\min\left\{k_{i},k_{j}\right\}+1-a_{ij}}\left(u\neq i,j\right)$). The dissimilarity matrix D (*d*_*ij*_ = 1−*TOM*_*ij*_) was then constructed, and hierarchical clustering was carried out for this matrix. The dynamic cutting tool in WGCNA was used to prune the cluster tree, and the gene modules were preliminarily divided. Based on the similarity of the eigengenes of the original modules, these modules were combined to form the final modules.

### Identification of the module and hub genes related to cashmere development

As shown in our previous publication (18), the cycle of cashmere growth could be divided into three distinct periods: a growth period (March–September), a regression period (September–December) and a resting period (December–March). March was considered to be the beginning of the cycle. In this study, among all 14 co-expression modules, the expression pattern of the blue module from 1 to 12 months was consistent with the growth cycle of cashmere. The expression pattern of the blue module gradually increased beginning in the growth period and showed a downward trend in the regression period and resting period. Moreover, after melatonin implantation, cashmere goats exhibited cashmere growth in May, which was also consistent with the blue module expression pattern. Therefore, the blue module was selected for relevant analysis in this study. The protein–protein interaction (PPI) network of the differentially expressed genes in the blue module was predicted using the Search Tool for the Retrieval of Interacting Genes/Proteins (STRING, version 11.5) (19), and a comprehensive score >0.4 was considered to indicate a statistically significant difference. The results of STRING analysis were imported into Cytoscape (20) to visualize the interaction network. The maximal clique centrality (MCC) metric was used to identify hub genes in the PPI network. MCC performed better than CytoHubba (21) in 11 other available methods (22), and the genes with the top 15 MCC scores were considered hub genes.

### Enrichment analysis of key module genes

To explore the biological functions of the differentially expressed genes, Gene Ontology (GO)/Kyoto Encyclopedia of Genes and Genomes (KEGG) enrichment analyses were performed on the differentially expressed genes between the implanted group and the control group in May. GO enrichment analysis was performed using the online tool g: Profiler (23). The GO categories included biological process (BP), cellular component (CC), and molecular function (MF). Significantly enriched GO terms were selected according to *P* < 0.05. The goat database was selected for KEGG pathway enrichment analysis with KOBAS 3.0 (24), and *P* < 0.05 was set as the screening criterion for significant enrichment.

### Combined analysis of hub genes and DEGs

To identify the genes expressed in May that play a role in activating cashmere growth in advance of the seasonal cycle, we screened for the genes differentially expressed in May between the implantation group and the control group. The thresholds for differential gene expression were | log2-fold change | > 1 and *P* < 0.05. Then, Venny 2.1.0 was used to determine the overlap among the three sets of genes.

### Quantitative real-time PCR

In this study, an Applied Biosystems QuantStudio 3 real-time fluorescence quantitative PCR system was used to reverse transcribe the extracted total RNA into cDNA according to the instructions of a PrimeScript™ RT Master Mix Kit (RR036A, TAKARA). Then, based on the cDNA sequences of the goat *PDGFRA, WNT5A, BMPR2*, and *BMPR1A* genes published by NCBI, specific primers were designed with Primer 5.0 (**Table 3**) and synthesized by Shanghai Bioengineering Co., Ltd. Finally, a TB Green “Premix Ex Taq” II Kit (RR820A, TAKARA) was used for qRT–PCR. Six samples were tested for each month, and three technical replicates were performed.

### Statistical analysis

The 2^−△△^*CT* method was used for the analysis of all qRT–PCR data, and SPSS software (version 22.0) was used for statistical analysis. Values are expressed as the means ± standard deviations. A significance level of 0.05 was used.

## Results

### Histological examination of goat skin

**Figure 1:**
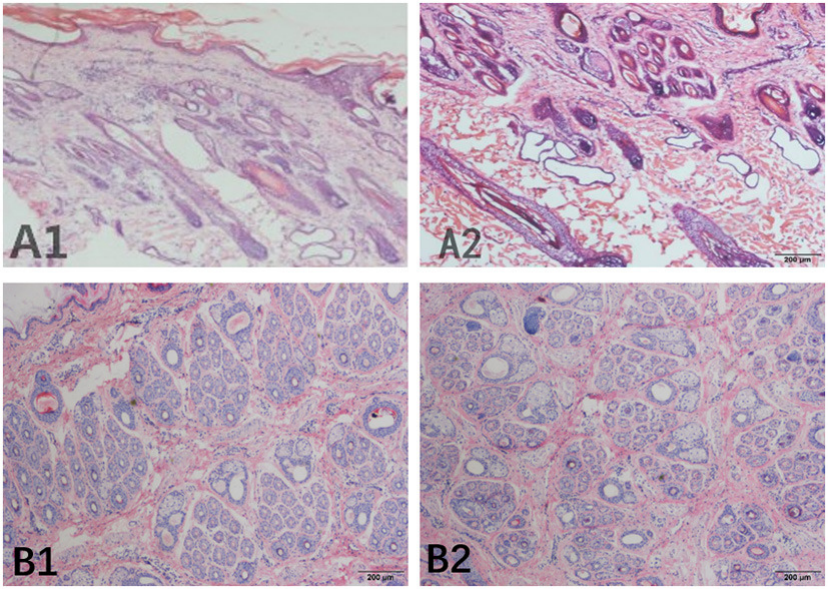
Skin tissue section of Inner Mongolia cashmere goats collected in May. **(A)** Represents the longitudinal section, **(B)** represents the transverse section, and numbers 1 and 2 represent the implanted group and the control group, respectively.

The S:P (ratio of secondary hair follicles to primary hair follicles) was used as an indicator of the number of hair follicles in the skin of the cashmere goats. In this study, we performed histological staining of sample sections from the control group and the implanted group in May (Figure 1), and six fields of view were observed for each sebaceous gland sample collected at different depths. The number of hair follicles in each counting area was calculated, and the results showed that the hair follicles of the control group were located deeply in the epidermal layer, while the hair follicles of the implanted group were located near the epidermal layer. The ratio of secondary hair follicles to primary hair follicles in the implanted group was significantly higher than that in the control group (*P* < 0.01).

### Quality of sequencing data and splicing results

The sequencing data for the 24 samples are shown in Table 1. The Trinity and Velvet assembly methods were used for comparison. When the Velvet assembly method was used, the effect was best when the kmer (bp) value was 57; 323,630 transcripts were obtained, and 67.27% of the transcripts were successfully mapped to the genome. With the Trinity method, 511,110 transcripts were obtained, and 90.06% of the transcripts were successfully mapped to the goat genome. Comparison of the genome mapping results indicated that the Trinity method performed better.

**Table 1:** Results of high-quality raw data.

**Sample name**	**Number of reads**	**Total length (bp)**	**Percentage (%)**
LZH-1	11901322	2088403976	86.87
LZH-2	11799342	2093444071	87.83
LZH-3	13129996	2254870449	85.02
LZH-4	12177094	2048134833	83.27
LZH-5	12151639	2130929746	86.81
LZH-6	11984821	2104532776	86.93
LZH-7	15948330	2710213186	84.13
LZH-8	11420123	2016743197	87.42
LZH-9	12990697	2298341553	87.59
LZH-10	12851175	2269169813	87.41
LZH-11	15025883	2513464188	82.81
LZH-12	13299627	2221588767	82.69
LZH-A	21997027	3736758621	84.10
LZH-B	17376784	2939183676	83.73
LZH-C	19578410	3294127588	83.29
LZH-D	13946831	2350977514	83.45
LZH-E	10365856	1623233029	77.52
LZH-F	13348217	2233152344	82.82
LZH-G	18423325	3104576795	83.42
LZH-H	13380685	2283690673	84.49
LZH-I	11977439	2114244179	87.39
LZH-J	12751089	2262360512	87.83
LZH-K	12775926	2249682980	87.17
LZH-M	13890435	2452180440	87.39

### Construction of the weighted gene co-expression network

**Figure 2:**
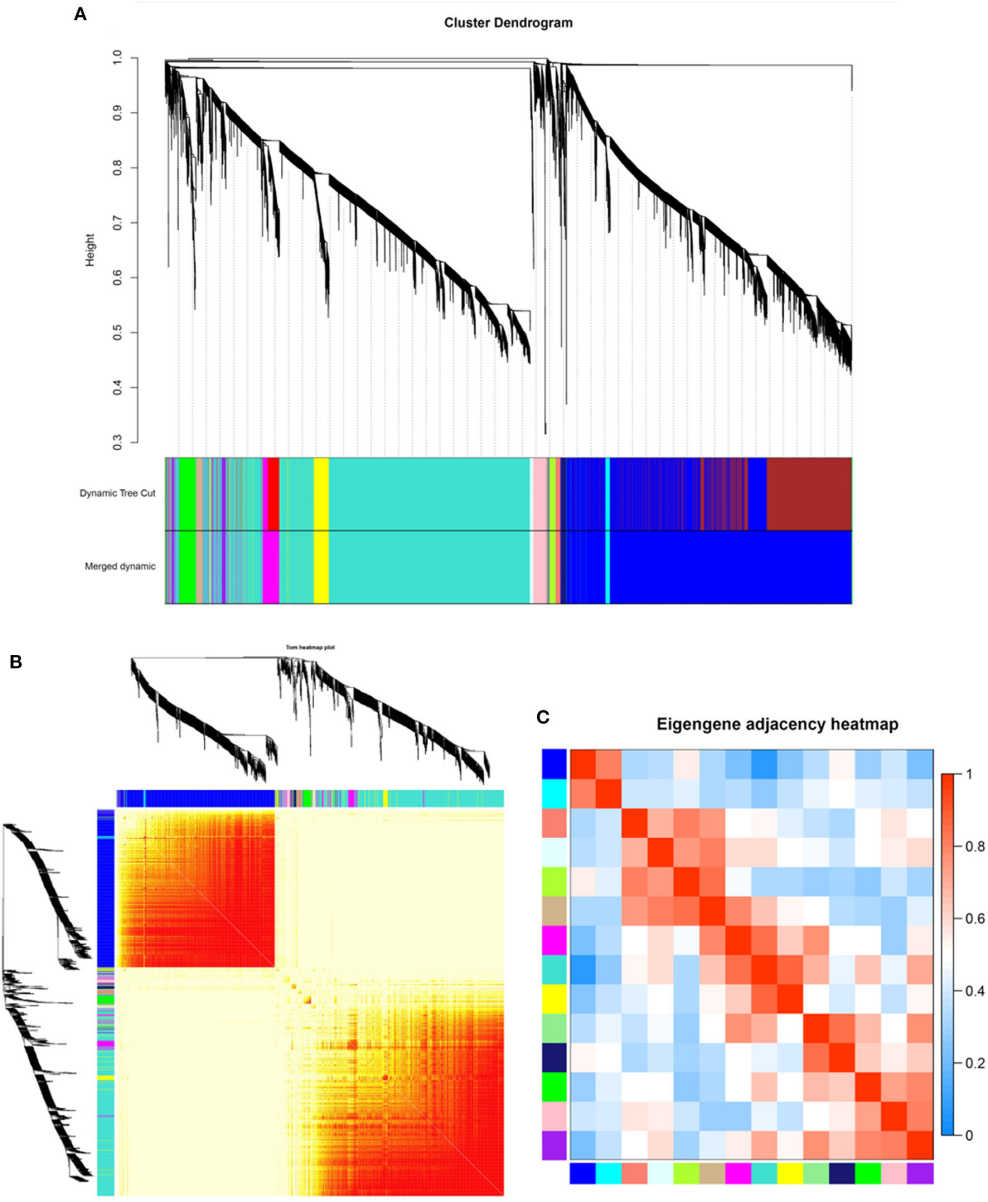
Identification of co-expression modules by WGCNA. **(A)** Dendrogram of gene clustering. **(B)** Network heatmap of module genes. Each tree represents a module, and each branch represents a gene. The darker the color of each dot is, the stronger the connectivity between the two genes in the row and column. **(C)** Correlation heatmap of each module; colors indicate correlations between modules. The closer the color is red, the higher the correlation, and the closer the color is blue, the lower the correlation.

RNA-seq was performed on 12-month transcriptome data for goat skin samples from the implantation group and the control group. Average linkage hierarchical clustering based on the computed topological overlap was then used to identify genes with very similar expression patterns in each module. A total of 14 co-expression modules were identified in the WGCNA network and represented by different colors. The resulting blue, cyan, green, green–yellow, light cyan, light green, magenta, midnight blue, pink, purple, salmon, tan, turquoise, and yellow modules contained 4,530, 84, 291, 117, 61, 41, 402, 76, 212, 119, 103, 105, 4,574, and 301 genes, respectively (a total of 11,016 genes) (Table 2). In the gene clustering results, Dynamic Tree Cut represents each group of genes, and Merged Dynamic represents the merged tree obtained by the dynamic cutting method (Figure 2A). In the topological overlap heatmaps, more topological overlap was observed within the modules than across the modules (Figure 2B). Based on correlation coefficient analysis, we clustered the modules associated with cashmere growth and development (positive or negative) (Figure 2C).

**Table 2:** Number of genes in the co-expression module.

**Module color**	**Gene numbers**	**Module color**	**Gene numbers**
Blue	4530	Midnightblue	76
Cyan	84	Pink	212
Green	291	Purple	119
Greenyellow	117	Salmon	103
Lightcyan	61	Tan	105
Lightgreen	41	Turquoise	4574
Magenta	402	Yellow	301

**Table 3:** Primer sequences specific for down-producing goat *PDGFRA, WNT5A, BMPR2, BMPR1A* and *GAPDH* and PCR product size.

**Gene name**	**Sequence of primer**	**Products size**
*PDGFRA*	F: GAGTGCCATTGAGACAGGTTCCAG	143 bp
	R: CCGAATCTGCCAGTTACAGGAAGC	
*WNT5A*	F: TCCAAGATTCAAAGAGCCTGCTTCC	145 bp
	R: AGCGTCCACTCCTGCCTACTTC	
*BMPR2*	F: CAACACCACTCAGTCCACCTCATTC	139 bp
	R: CCTTGTTTGCGGTCTCCTGTCAG	
*BMPR1A*	F: CCAGGTCAGCAATACAGCAACTCC	112 bp
	R: CTCCACACAGAAATCTACGGCACTC	
*GAPDH*	F: TGGACATCGTTGCCATCAATGACC	100 bp
	R: TTGACTGTGCCGTGGAACTTGC	

### Identification of the module and hub genes related to cashmere development

**Figure 3:**
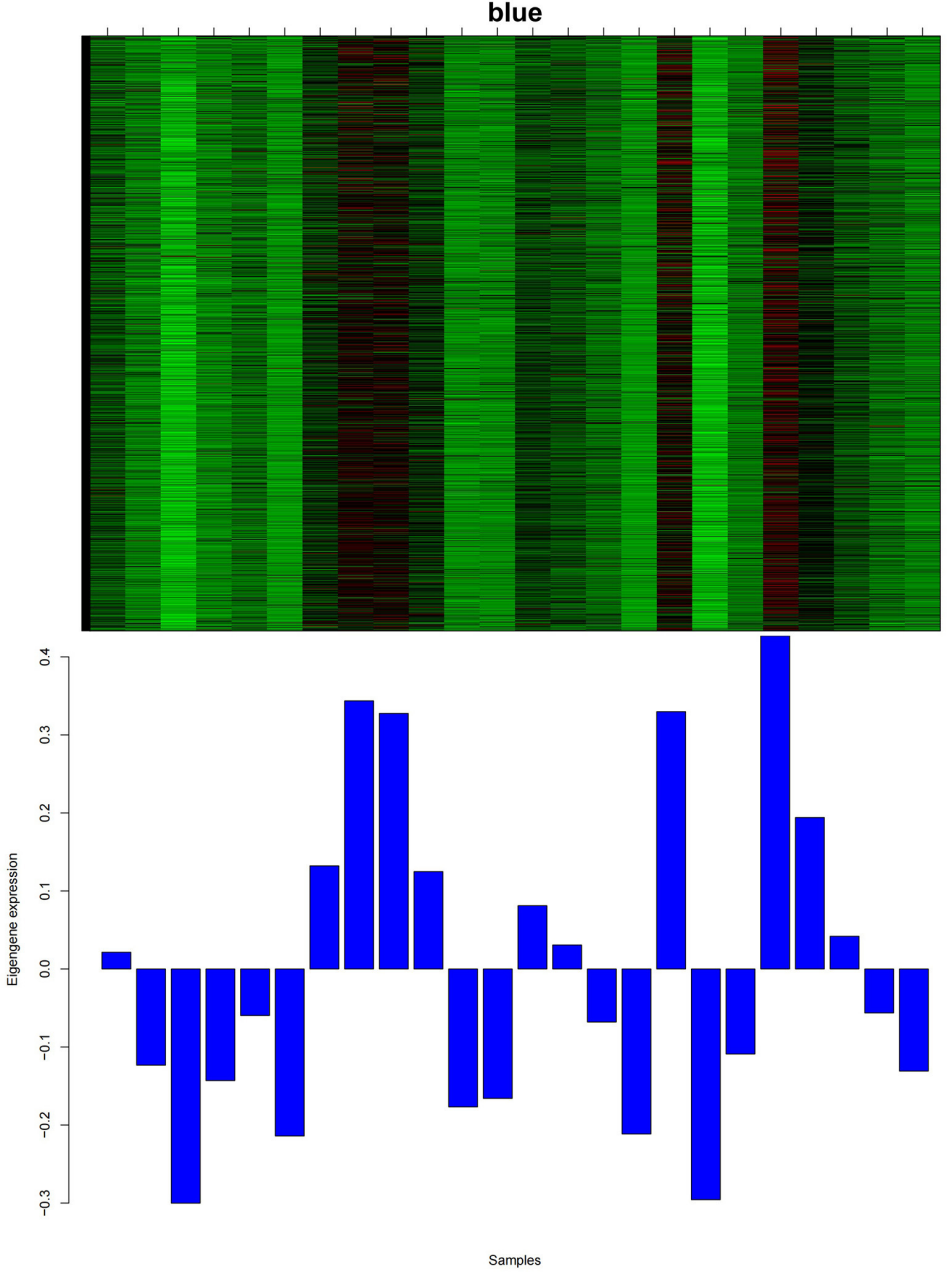
Heatmap of the gene expression pattern of the blue module. The abscissa is the name of the sample. The top figure shows the heatmap of gene expression in different samples of the module, and the bottom figure shows the expression pattern of the feature value of the module in different samples.

As shown in Figure 3, among all 14 co-expression modules, the expression pattern of the blue module from 1 to 12 months was consistent with the growth cycle of cashmere, as previously shown by our group. Moreover, after the implantation of melatonin, cashmere goats exhibited cashmere growth in May, which was also consistent with the blue module expression pattern. Therefore, the blue module was selected for relevant analysis in this study. We constructed PPI networks for the genes in the blue module. The genes with the top 15 MCC scores in CytoHubba were identified as hub genes.

### Enrichment analysis of key module genes

We further performed GO/KEGG enrichment analyses with 316 differentially expressed genes upregulated in the blue module in May. According to GO classification statistics, 148 terms were grouped into three GO categories: cellular component, molecular function, and biological process. Among the three GO categories, the most significantly enriched were cellular protein modification process, desmosome and protein binding. Notably, the cellular components were significantly enriched in desmosomes, which are the most important intercellular adhesion junctions, directly adhering to desmosomal cadherins on adjacent keratinocytes to form the epidermal layer.

**Figure 4:**
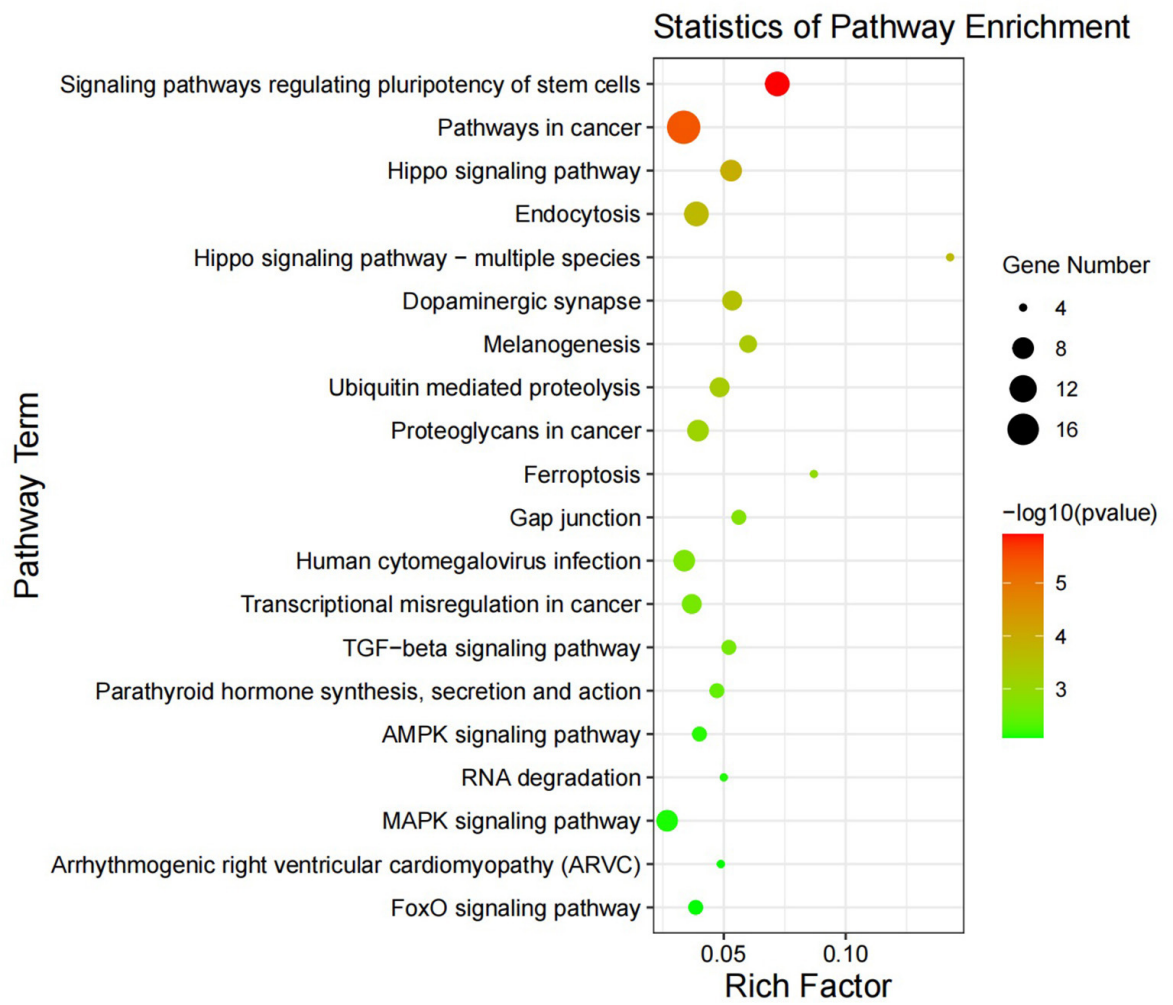
Differential gene KEGG enrichment diagram. The X-axis represents the ratio of enriched differential genes to the total number of genes in the term, and the Y-axis represents the enriched function/pathway. The colors represent the degree of enrichment significance; the closer the color is red, the more significant the results are. The circle size represents the number of genes enriched.

**Figure 5:**
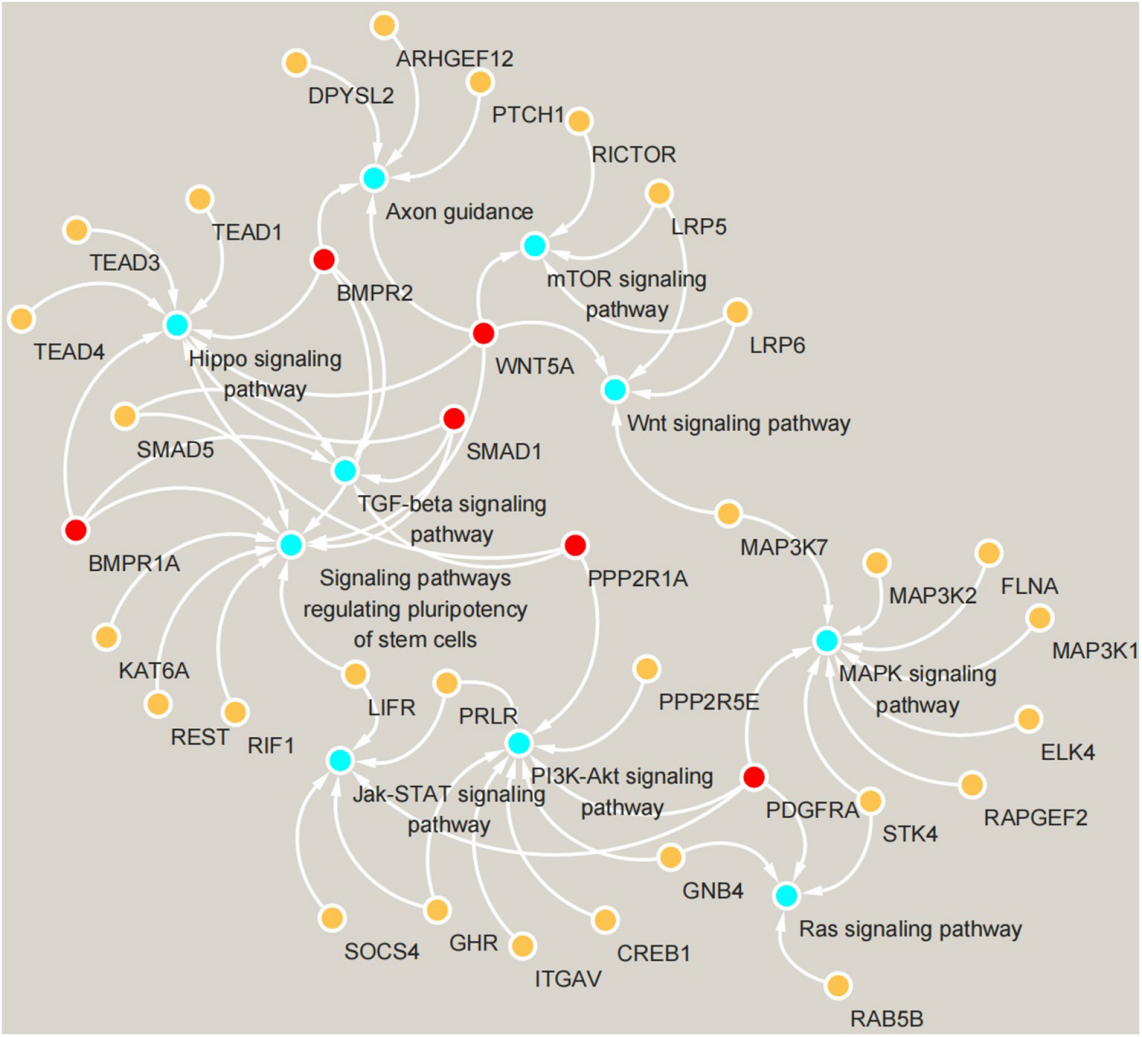
Gene enrichment map of KEGG signaling pathways. Blue circles represent signaling pathways, yellow circles represent genes enriched in pathways, and red circles represent genes enriched in more than three pathways simultaneously.

KEGG pathway analysis (Figure 4) showed that in May, the differentially expressed genes between the control group and the implanted group were mainly enriched in the signaling pathway that regulates the pluripotency of stem cells and the Hippo, TGF-beta and MAPK signaling pathways. Interestingly, we found significant enrichment of MT signaling pathways. According to the KEGG enrichment analysis results, six genes were enriched in three or more signaling pathways related to hair follicle development: *PDGFRA, WNT5A, BMPR1A, BMPR2, PPP2R1A*, and *SMAD1* (Figure 5).

### Combined analysis of hub genes and differentially expressed genes

**Figure 6:**
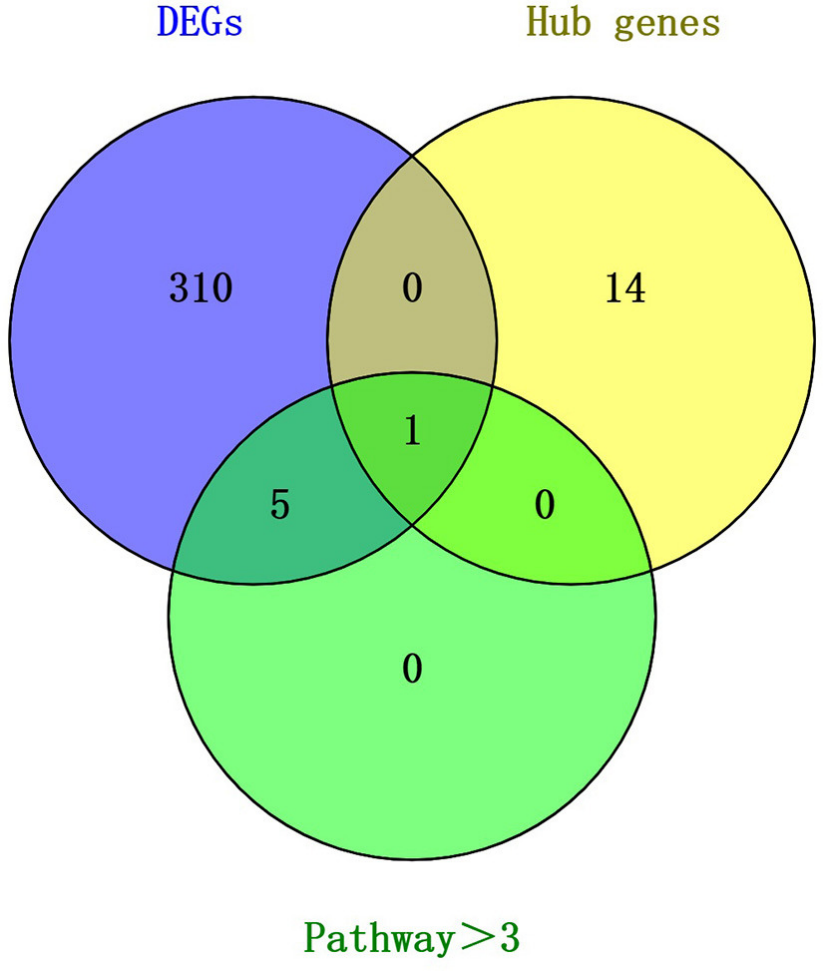
Venn diagram of key candidate genes.

We compared the 316 genes differentially upregulated in the control group and the implanted group in May with the important genes and hub genes in the PPI. *PDGFRA* was common to all three gene sets (Figure 6). This result suggests that *PDGFRA* is a key gene in the early growth of cashmere after implantation of MT.

### The relative expression of core genes was determined by qRT–PCR

**Figure 7:**
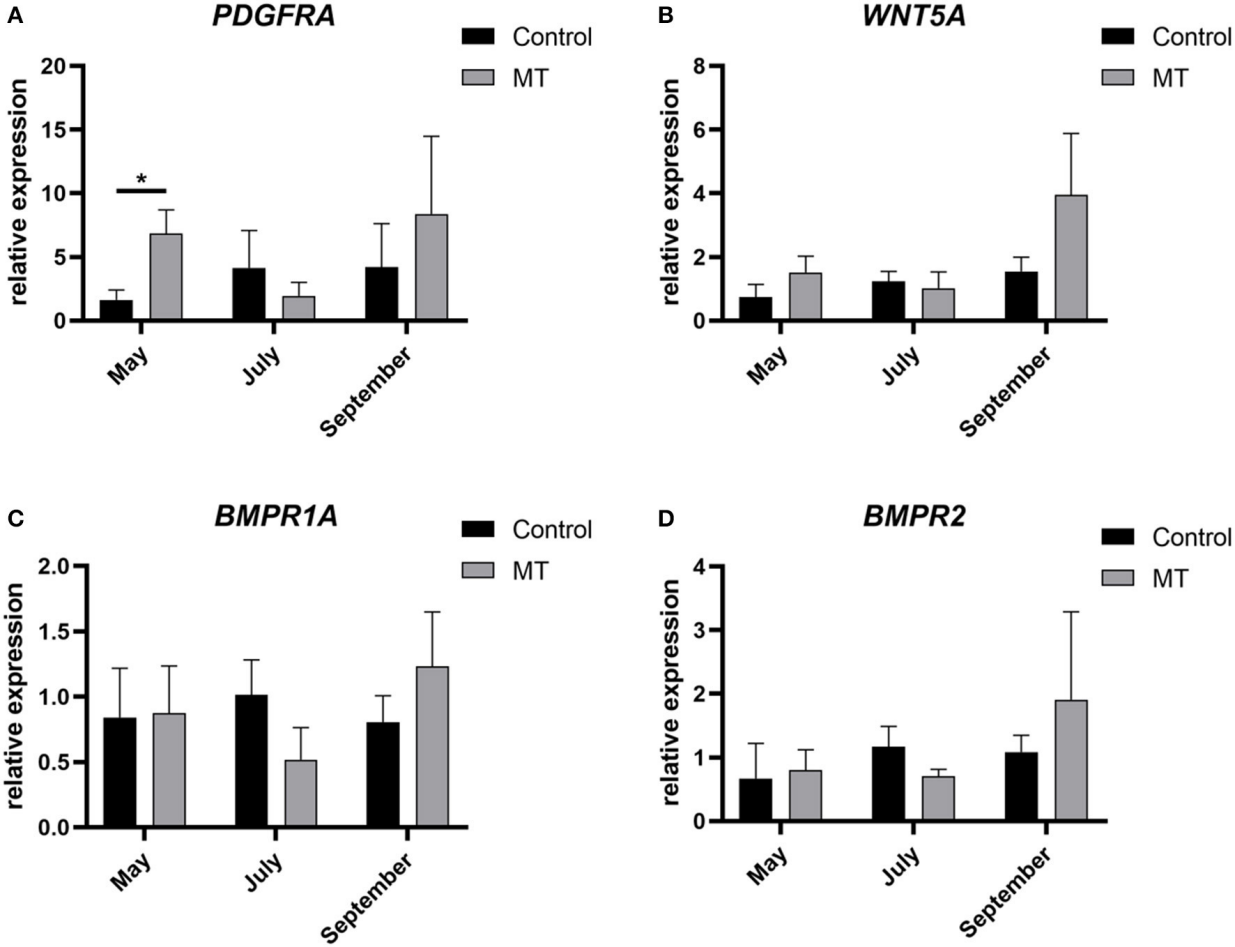
Sequencing results validated by fluorescent qRT-PCR. **(A–D)** The relative expression of four genes. The abscissa represents month, the ordinate represents relative expression, *Represents a significant difference between groups (*P* < 0.05), and the error bar represents the SD.

In this study, total RNA was extracted from skin samples of the control group and the implanted group at each of the 12 months. A NanoDrop 2000 UV spectrophotometer was used to determine whether the OD260/280 values were between 1.8 and 2.0. Four genes were randomly selected to evaluate their relative expression with respect to *GAPDH* as the internal reference gene to verify the accuracy of the sequencing results. The qRT–PCR results were basically consistent with the transcriptome data. The expression levels of *PDGFRA, WNT5A, BMPR2*, and *BMPR1A* in the control group showed a gradually increasing trend from May to September but first decreased and then increased in the implanted group. The *PDGFRA* level differed significantly (*P* < 0.05) between the control group and the implanted group in May (Figure 7). This finding is consistent with our analysis results: *PDGFRA* is a key gene in the promotion of premature cashmere production by MT implantation.

## Discussion

The main objective of this study was to screen for and identify the genes related to early hair production after MT implantation in Inner Mongolia cashmere goats and to study the role of these genes in the growth and development of skin hair follicles in Inner Mongolia cashmere goats. According to the longitudinal analysis of sections of samples from the implanted group and control group that were collected in May, the hair follicles of the control group were located deeply in the epidermal layer, while the hair follicles of the implanted group were located near the epidermal layer, indicating that the hair follicles in the melatonin-implanted group emerged sooner than those in the control group. Through the analysis of cross-sections, it was found that the ratio of secondary hair follicles to primary hair follicles in the implanted group was significantly higher than that in the control group, indicating that the growth of secondary hair follicles in the implanted group was earlier than that in the control group. On the basis of transcriptome sequencing data, we identified 14 co-expression modules using WGCNA, selected the blue module as having an expression that aligned with the growth cycle of cashmere for analysis, and constructed a predicted protein interaction network. Key genes within the blue module were identified using the STRING database and CytoHubba. GO and KEGG analyses were performed with the differentially expressed genes. The upregulated differentially expressed genes were compared with hub genes and the genes enriched in 3 or more signaling pathways in the implanted group and the control group in May, providing important insights into the transcriptional mechanism of MT-mediated skin hair follicle development in Inner Mongolia cashmere goats.

Genes play a decisive role in the process of cashmere growth. Previous studies have found that implanting MT can promote the early growth of cashmere, but the specific genes and pathways involved in the early growth of cashmere remain to be studied. Therefore, it is highly important to study the genes related to cashmere growth regulation after MT implantation. The pathway enrichment analysis in this study identified six genes enriched in more than three pathways: *PDGFRA, WNT5A, PPP2R1A, BMPR2, BMPR1A*, and *SMAD1*. The signaling pathway regulating the pluripotency of stem cells was the pathway most significantly enriched with the differentially expressed genes. This result may indicate that stem cells play an important role in hair follicle development during the hair cycle, but no relevant reports have been made. Moreover, differentially expressed genes were also enriched in the Hippo, TGF-beta and MAPK signaling pathways, which are all related to cashmere growth (25–29).

In our previous studies, we found that the development of hair follicles is regulated by hair follicle stem cells, and through single-cell analyses, we found that hair follicle stem cells can affect the development cycle of cashmere. The epidermis regenerates in a steady state through balanced proliferation of basal cells and exfoliation of keratinized squamous cells on its surface. Hair follicles regenerate through a complex cyclical process. The cycle begins with the activation of stem cells, which is followed by the proliferation and differentiation of their progeny (30). *WNT5A*, which is critical to the initiation of hair growth, was enriched in the signaling pathway pluripotency of stem cells and the WNT signaling pathway (31–33). Wnt-secreted proteins can stimulate diverse signaling pathways in cells and play important roles in cell proliferation, differentiation and migration as growth regulators. Many studies have confirmed that the classical Wnt/β-catenin signaling pathway plays a key role in hair follicle growth (34). *WNT5A* can activate both classical and non-classical WNT signaling pathways and act through different WNT pathways after binding to different receptors in different cell types. The binding of *WNT5A* to the *FZD-4* receptor activates the β-catenin pathway to promote cell proliferation (35). Therefore, we hypothesized that *WNT5A* may affect the initiation of cashmere growth through activation of the β-catenin pathway.

A main role of the platelet-derived growth factor (PDGF) gene is to promote DNA synthesis in cells. PDGF participates in the regulation of hair follicle growth and development mainly through PDGF signaling (including through the *PDGFA* gene and PDGF receptors A and B). Studies have shown that PDGF signaling is related to hair follicle morphogenesis in early hair follicle development and that inhibition of the expression of PDGF or its receptor *PDGFRA* or *PDGFRB* in growing and developing hair follicles prevents hair follicle maturation (36). In this study, *PDGFRA* was identified as a key gene in four pathways, and many genes in the blue module are related to *PDGFRA*, such as *IGF1, ITGAV, FRS2*, and *CDK6*. Most of these genes are related to cell growth, and some researchers have found that the treatment of acute wounds in rats with *IGF1*-overexpressing fibroblasts can significantly improve the rate of wound healing (37). *ITGAV* has been proven to be a key gene for the initiation of goat cashmere growth (38). Cyclin-dependent kinase 6 (*CDK6*) is an important regulator of the cell cycle and plays a role to similar *CDK4* as a mediator of keratinocyte proliferation (39). The docking protein *FRS2* is involved in the transmission of extracellular signals from fibroblast growth factor (FGF) or nerve growth factor (NGF) receptors to the Ras/MAPK signaling pathway (40). The MAPK and PI3K-AKT signaling pathways have been confirmed to play an important role in the initial stage of cashmere development (25, 41). The Jak-stat signaling pathway may be involved in the transition from the resting to the growth phase in cashmere growth (42), and the Ras signaling pathway is also believed to be involved in the development and regeneration of hair follicles (43). Early research by our group through simple correlation analysis of the expression levels of cashmere growth-related genes showed that each signaling pathway was interconnected and interwoven into a network to regulate the periodicity of cashmere growth. MT implantation can promote the expression of *PDGFA* and its receptor, *PDGFRA*, as well as their binding. *PDGFA, PDGFRA*, and *NTRK3* play a synergistic role in the periodic growth of cashmere. During robust growth, the PDGF signaling pathway plays an important role, consistent with the results of this study.

At the beginning of the growth phase, follicular progenitor cells in the epidermis induce mesenchymal condensation to form the dermis, which then produces proliferating stromal cells. These epidermal cells further differentiate into hair stem cells; in addition, some of these stem cells form the basal layer of the epidermis. The BMP family is the largest subfamily of growth factors in the TGF-beta superfamily and controls hair follicle morphogenesis at many different stages (44). BMP signaling occurs through a complex of type I (BMPRI) and type II (BMPRII) BMP receptors. *BMPR1A* is one of the three type I BMP receptors. Deletion of the *BMPR1A* gene in mouse skin hair follicles resulted in reduced hair follicle differentiation and interfollicular epidermal cell growth in mouse fetal skin and in a significant reduction in the number of hair follicles in postnatal mice, which were hairless in the affected area (45). These results suggest that *BMPR1A* signaling is critical not only for hair follicle differentiation during development but also for epidermal cell proliferation or differentiation during the renewal of the adult cashmere cycle. In addition, TGF-beta signaling plays a key role in various aspects of hair follicle development and circulation (44), and some experimental studies have demonstrated that *BMPR2* is essential for normal hair development and maintenance. Decreased *BMPR2* expression results in premature end of the growth phase. This finding also confirms the positive effect of *BMPR2* on the initiation and maintenance of cashmere in this study.

Mammalian hair follicle development begins in the embryonic stage, and studies have shown that Wnt/β-catenin, TGF-beta/Smad and other signaling pathways are involved in the early initiation, differentiation and development of the hair follicle tissue structure (46). In skin hair follicle tissue, *SMAD1* mainly plays a role in promoting tissue differentiation and hair follicle formation; in contrast, *SMAD1* is rarely expressed in the base of mature hair follicles. Therefore, we speculated that *SMAD1* may be a key promoter gene of cashmere development. *PPP2R1A* is the scaffold subunit of protein phosphatase 2A (PP2A), one of the four major serine/threonine protein phosphatases. The mechanism of this gene in hair follicle development in cashmere goats has not been reported, but based on the signaling pathways and increased expression level of *PPP2R1A*, we speculate that *PPP2R1A* may be related to cell proliferation and differentiation, a possibility that will constitute our future research direction. In general, in this study, by using WGCNA, we identified 6 key genes that may mediate the effects of MT and lead to early cashmere production in cashmere goats, providing a foundation for subsequent research.

## Conclusion

In this study, WGCNA was used to investigate the mechanism by which exogenous melatonin regulates the cycle of cashmere developmental. Histological sections of goat skin that were collected in May showed that the hair follicles of the control group were located deeply in the epidermal layer, while the hair follicles of the implanted group were located near the epidermal layer. The ratio of secondary hair follicles to primary hair follicles in the implanted group was significantly higher than that in the control group. A total of 14 co-expression modules were identified, and the blue module was found to be correlated with the cashmere growth cycle after MT implantation. Through analysis of the hub genes, the genes in the blue module and the genes differentially expressed between the implanted group and the control group in May, *PDGFRA* was identified as the key gene through which MT regulates cashmere growth, providing a theoretical basis for further improving cashmere yield and economic benefit.

## Data availability statement

The datasets presented in this study can be found in online repositories. The names of the repository/repositories and accession number(s) can be found below: https://www.ncbi.nlm.nih.gov/, SRP145408.

## Ethics statement

In this study, skin samples were collected in accordance with the International Guiding Principles for Biomedical Research involving animals, and the experiment was approved by the Special Committee on Scientific Research and Academic Ethics of Inner Mongolia Agricultural University, which is responsible for the approval of Biomedical Research Ethics of Inner Mongolia Agricultural University [Approval No. (2020) 056]. No specific permissions were required for these activities, and no endangered or protected species were involved (47).

## Author contributions

ZhihL and YZhao contributed to the concept and design of the study. ZhicL, MZ, TC, ZhixW, CZhao, CZhan, QQ, XX, and ML collected the samples and extracted the RNA. QM performed the sections and staining. ZhicL, YX, and QQ conducted the data analysis. YZhan, RS, ZhiyW, RW, and JL provided technical support. ZhicL and ZhihL wrote the first draft of the manuscript. All the authors contributed to the revision of the manuscript and read and approved the submitted version.

## Funding

This work was supported by the National Key R&D Program of China (2021YFD200901); the National Natural Science Foundation of China (32160772, 32060742, and 31860628); the Major Science and Technology Projects of Inner Mongolia Autonomous Region (2020ZD0004); the Key Technology Project of Inner Mongolia Autonomous Region (2020GG0030 and 2020GG0031).

## Conflict of interest

The authors declare that the research was conducted in the absence of any commercial or financial relationships that could be construed as a potential conflict of interest.

## Publisher's note

All claims expressed in this article are solely those of the authors and do not necessarily represent those of their affiliated organizations, or those of the publisher, the editors and the reviewers. Any product that may be evaluated in this article, or claim that may be made by its manufacturer, is not guaranteed or endorsed by the publisher.
